# PeakForce Tapping resolves individual microvilli on living cells†

**DOI:** 10.1002/jmr.2510

**Published:** 2015-09-28

**Authors:** Hermann Schillers, Izhar Medalsy, Shuiqing Hu, Andrea L. Slade, James E. Shaw

**Affiliations:** ^1^ Institute of Physiology IIUniversity of MünsterRobert‐Koch‐Str. 27bMünster48149Germany; ^2^ Bruker Nano Surfaces Division112 Robin Hill RdSanta BarbaraCA93117USA

**Keywords:** AFM, low‐force imaging, live cell AFM probe, MDCK, Peakforce Tapping

## Abstract

Microvilli are a common structure found on epithelial cells that increase the apical surface thus enhancing the transmembrane transport capacity and also serve as one of the cell's mechanosensors. These structures are composed of microfilaments and cytoplasm, covered by plasma membrane. Epithelial cell function is usually coupled to the density of microvilli and its individual size illustrated by diseases, in which microvilli degradation causes malabsorption and diarrhea. Atomic force microscopy (AFM) has been widely used to study the topography and morphology of living cells. Visualizing soft and flexible structures such as microvilli on the apical surface of a live cell has been very challenging because the native microvilli structures are displaced and deformed by the interaction with the probe. PeakForce Tapping® is an AFM imaging mode, which allows reducing tip–sample interactions in time (microseconds) and controlling force in the low pico‐Newton range. Data acquisition of this mode was optimized by using a newly developed PeakForce QNM‐Live Cell probe, having a short cantilever with a 17‐µm‐long tip that minimizes hydrodynamic effects between the cantilever and the sample surface. In this paper, we have demonstrated for the first time the visualization of the microvilli on living kidney cells with AFM using PeakForce Tapping. The structures observed display a force dependence representing either the whole microvilli or just the tips of the microvilli layer. Together, PeakForce Tapping allows force control in the low pico‐Newton range and enables the visualization of very soft and flexible structures on living cells under physiological conditions. © 2015 The Authors Journal of Molecular Recognition Published by John Wiley & Sons Ltd.

## Introduction

Microvilli are soft and flexible membrane protrusions of epithelial cells extending to the luminal surface in lung (Krasteva and Kummer, 2012), intestine (Helander and Fandriks, 2014), kidney (Weinbaum *et al*., 2010), and other organs (Dickersin, 1987; Makabe *et al*., 2006; Sekerkova *et al*., 2006; Sundd *et al*., 2011). A microvillus is shaped cylindrical with a length of 1–2 µm and a diameter of 50–100 nm (McConnell *et al*., 2009). It consists of plasma membrane‐covered actin bundles stabilized by cross‐linking proteins (Loomis *et al*., 2003). The function of microvilli is directly related to their structure. Enlargement of the apical cell surface by microvilli could be 100‐fold compared with a flat surface. This enhances vectorial transcellular transport such as absorption and secretion and also increases strongly the capacity to house membrane‐bound transport proteins. The flexibility of these plasma membrane‐covered microfilaments allows the detection of fluid dynamics by transmitting microvillus bending to the actin cytoskeleton. This mechanosensory function regulates flow‐dependent Na^+^ absorption in proximal tubule (Du *et al*., 2004). Some epithelial cells have large numbers of microvilli that form a brush border, such as that found in the bronchial epithelium, small intestine, and renal tubules. Changes of microvilli density and morphology in some diseases can occur because of a rearrangement of host cells actin cytoskeleton (Hecht *et al*., 2011). This affects epithelial function, which leads to clinical manifestations like enteropathies such as congenital microvillus atrophy (Cutz *et al*., 1989) and Celiac disease (Bailey *et al*., 1989). Observing structural (and therefore functional) integrity of microvilli on living cells would help to understand the development of microvilli‐dependent diseases.

Atomic force microscopy (AFM) is a technique that has been widely used to image surfaces of live cells and has been used to resolve microvilli on living and fixated cells (Braet *et al*., 1998; Braet *et al*., 2001; Poole *et al*., 2004; Deng *et al*., 2010; Koehne *et al*., 2011). Identification of individual microvilli was possible on fixated cells, but the spatial resolution was not sufficient on live cells. When using contact or tapping mode, the topographic images are usually blurred. Microvilli are soft and flexible and are therefore easily displaced by forces applied during the scanning process. During scanning in contact mode or TappingMode, the probe applies vertical and lateral forces to the cell surface. The vertical force is needed to feel the surface, but the lateral forces are usually unwanted as they often cause blurring in the image because of dragging and pushing of flexible surface structures such as microvilli. Also, the vertical forces lower resolution because the minimum force required to bend the cantilever is sufficient to deform very soft structures resulting in an uncertainty of height and shape (Le Grimellec *et al*., 1998). Obviously, reducing vertical and lateral forces are necessary to improve imaging of microvilli. Even in TappingMode, reduction and localization of the vertical interaction require use of small amplitudes, which leads to lateral dragging through the spatially extended, soft structures, presented by the cell surface. PeakForce Tapping® mode (Pettinger *et al*., 2010) was developed to control vertical forces in the range of some tens of pico‐Newtons (pN). This AFM imaging mode is characterized by vertical oscillation of the probe far below its resonance frequency. Oscillation is driven in a sinusoidal waveform with amplitudes of typically 100–300 nm resulting in force–distance curves for each image pixel. The maximum probe–sample interaction force (peak force) of each curve is used to control vertical forces. Force Volume mode is another technique using force–distance curves to image soft samples (Hassan *et al*., 1998). Images are reconstructed by using the *z*‐piezo position at the moment the tip touches the surface (zero‐force image). While PeakForce Tapping and Force Volume modes are both essentially force–distance force curve imaging modes, PeakForce Tapping offers advantages over Force Volume for high‐resolution imaging of living cells. The combination of sinusoidal modulation and background subtraction conducted for each probe–sample interaction provides PeakForce Tapping with highly sensitive force control, which allows the use of low imaging forces. PeakForce Tapping is also capable of operating at frequencies of up to 1 kHz on live cells as compared with Force Volume, which has shown a maximum frequency of ~100 Hz. As Force Volume uses a triangular motion to move the probe in and out of contact with the sample surface, it has difficulty maintaining low set points or imaging forces at higher frequencies. Because of the short delay in the feedback loop from the time that the set point (trigger force) is reached and the time that the piezo changes its motion to move the AFM probe away from the sample, this can result in higher forces (overshoot) than the actual set point being applied to the sample at high frequencies. So, even though the zero‐force image is intended to show the cell topography at the moment the tip touches the cell surface in Force Volume mode, the actual force that the probe is applying to the cell is much higher and can possibly distort or effectively change the apparent structure of the cell surface. PeakForce Tapping mode has been successfully applied to image various delicate biological samples (Dufrene *et al*., 2013) like cells (Berquand *et al*., 2010; Heu *et al*., 2012; Pletikapić *et al*., 2012), membrane proteins (Medalsy *et al*., 2011; Rico *et al*., 2011; Alsteens *et al*., 2012), vesicles (Hardij *et al*., 2013), and amyloid fibrils (Adamcik *et al*., 2011). In the present work, we used PeakForce Tapping, which allows reduction of both vertical and lateral forces in order to achieve unrivaled resolution of microvilli on live epithelial cells.

## Materials and methods

### Cell culture

The Madin–Darby canine kidney (MDCK) cell subclone C11 resemble alpha‐intercalated cells (Gekle *et al*., 1994) and was grown at 37°C and maintained in modified minimum essential medium (MEM) containing Earl's balanced salt solution supplemented with 2 mM l‐glutamine, 10% heat‐inactivated fetal calf serum (FCS), 50 IU/ml penicillin, and 50 µg/ml streptomycin in a 5% CO_2_‐humidified incubator. Confluent cell layers were subcultured weekly by trypsinization. For AFM experiments, cells were seeded in a density of 500 000 cells on 50‐mm glass bottom Petri dishes (WillCo Wells, Amsterdam, the Netherlands) and cultured for 5 days in the aforementioned medium. Before measurement, the medium was exchanged against HEPES‐Ringer buffer (in millimolar: Hepes (*N*‐(2‐hydroxyethyl)piperazine‐*N*′‐2‐ethane sulfonic acid) 10, NaCl 122.5, KCl 5.4, MgCl_2_ 0.8, CaCl_2_ 1.2, NaH_2_PO_4_ 1, and glucose 5.5).

### Atomic force microscopy

Atomic force microscopy was performed in HEPES‐Ringer buffer at room temperature in PeakForce Tapping mode using a BioScope Resolve AFM (Bruker Nano Surfaces, Santa Barbara, CA, USA). A PeakForce QNM‐Live Cell (PFQNM‐LC) probe (Bruker AFM Probes, Camarillo, CA, USA) (tip length 17 µm, tip radius 65 nm, opening angle 15°) was used to image the cell surface. The spring constant of the cantilever was determined with a vibrometer (OFV‐551, Polytec, Waldbronn, Germany) and found to be 0.0611 N/m. The glass bottom Petri dish in which the MDCK cells were grown was held down through the use of vacuum that has been incorporated into the AFM sample plate while still allowing optical access to the sample from below. The use of vacuum reduces noise and eliminates the “drum” effect that is created when these thin‐bottomed petri dishes are positioned over the optical aperture in the sample stage. Images were taken at 384 × 384 pixels with a PeakForce Tapping frequency of 1 kHz and amplitude of 300 nm. Probe–sample contact time was about 200 μs each cycle. Automatic gain control was used to improve the feedback for surface tracking. Height sensor signal was used to display the cell surface image using Nanoscope Analysis v1.60 (Bruker Nano Surfaces, Santa Barbara, CA, USA).

## Results

**Figure 1:**
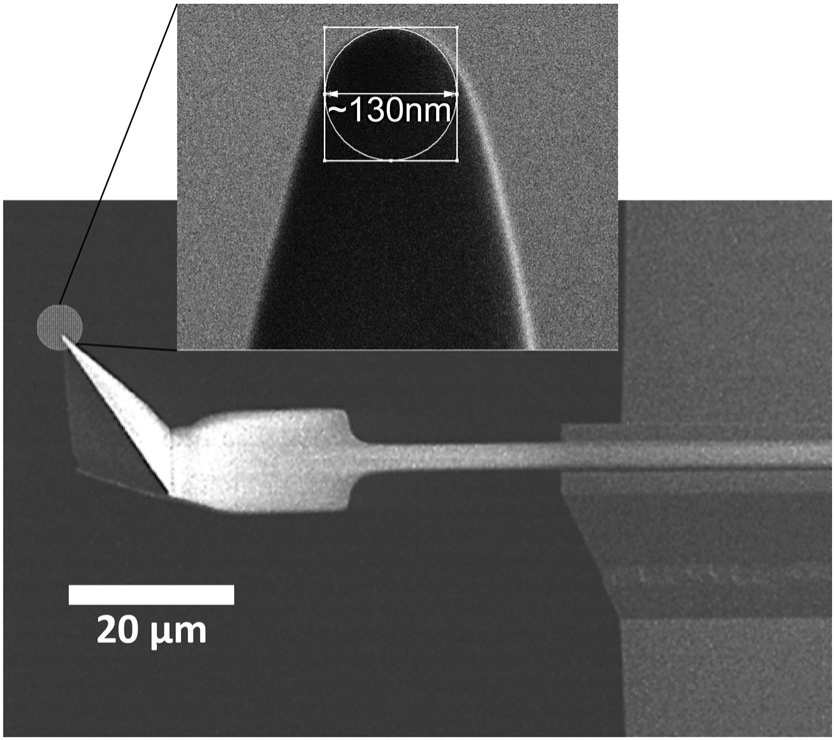
Scanning electron microscopy (SEM) image of the PeakForce QNM‐Live Cell probe. The 17‐µm tip is mounted on a paddle‐shaped 45‐µm‐long cantilever. The insert shows the sharpened end of the pyramidal tip having a length of 0.8–1 µm, a radius of 65 nm, and an opening angle of 15°.

**Figure 2:**
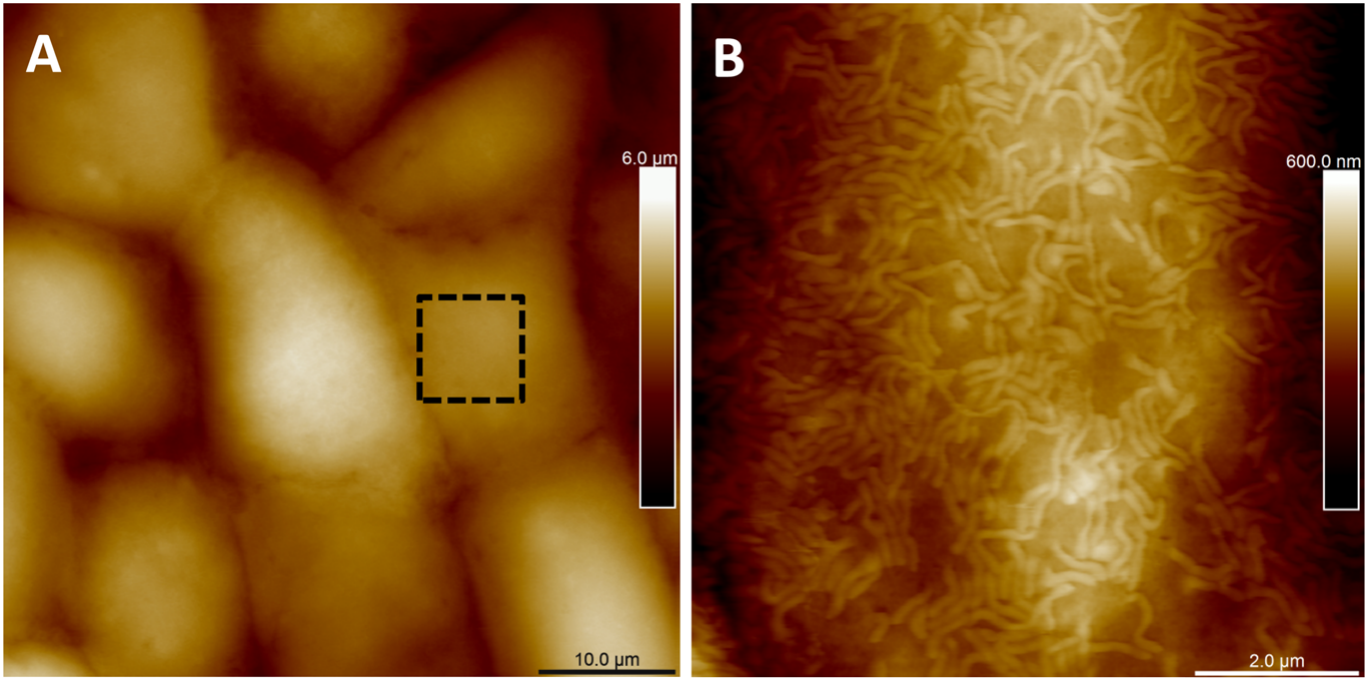
PeakForce Tapping image of glutaraldehyde‐fixed MDCK C11 cells. Overview scan (50 × 50 µm) of several cells (A) and a higher resolved image (8 × 8 µm) in the area indicated by the black outline (B).

**Figure 3:**
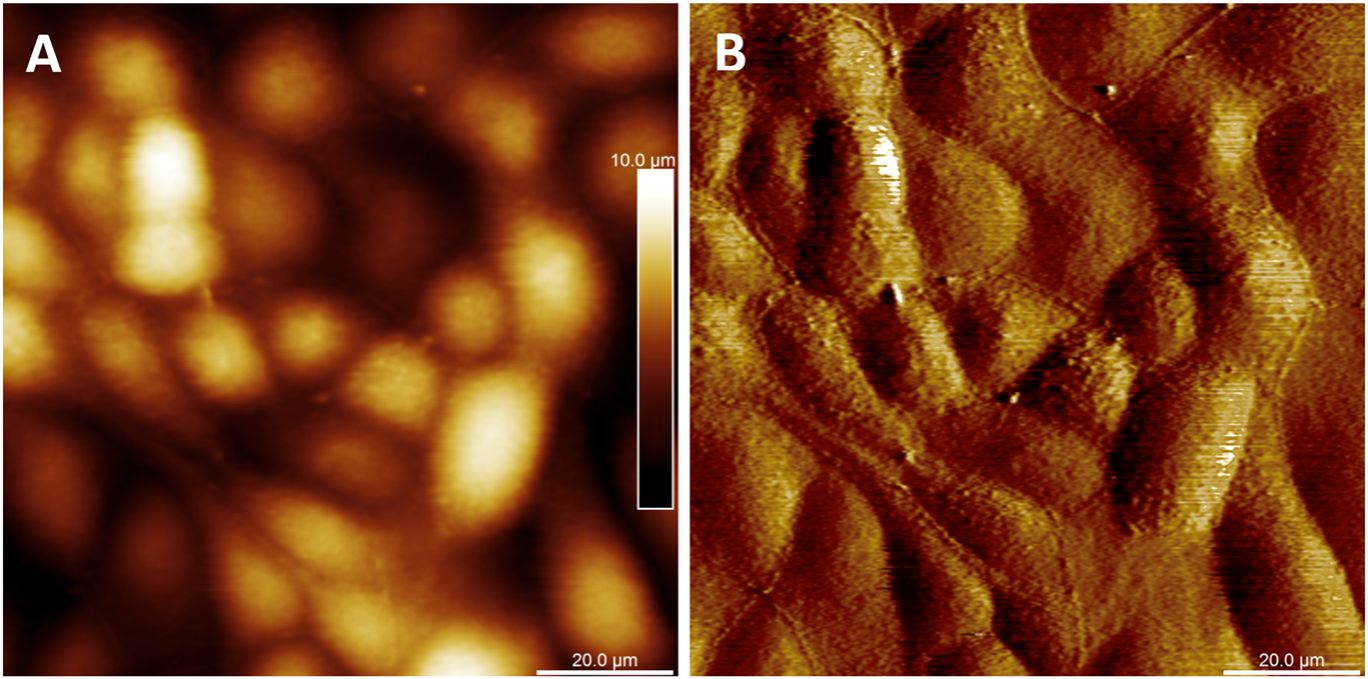
PeakForce Tapping image of an MDCK C11 monolayer. The left panel represents the height sensor image, and the right panel represents the error signal. Scan resolution is 384 lines with 384 samples per line (pixel size 260 nm).

**Figure 4:**
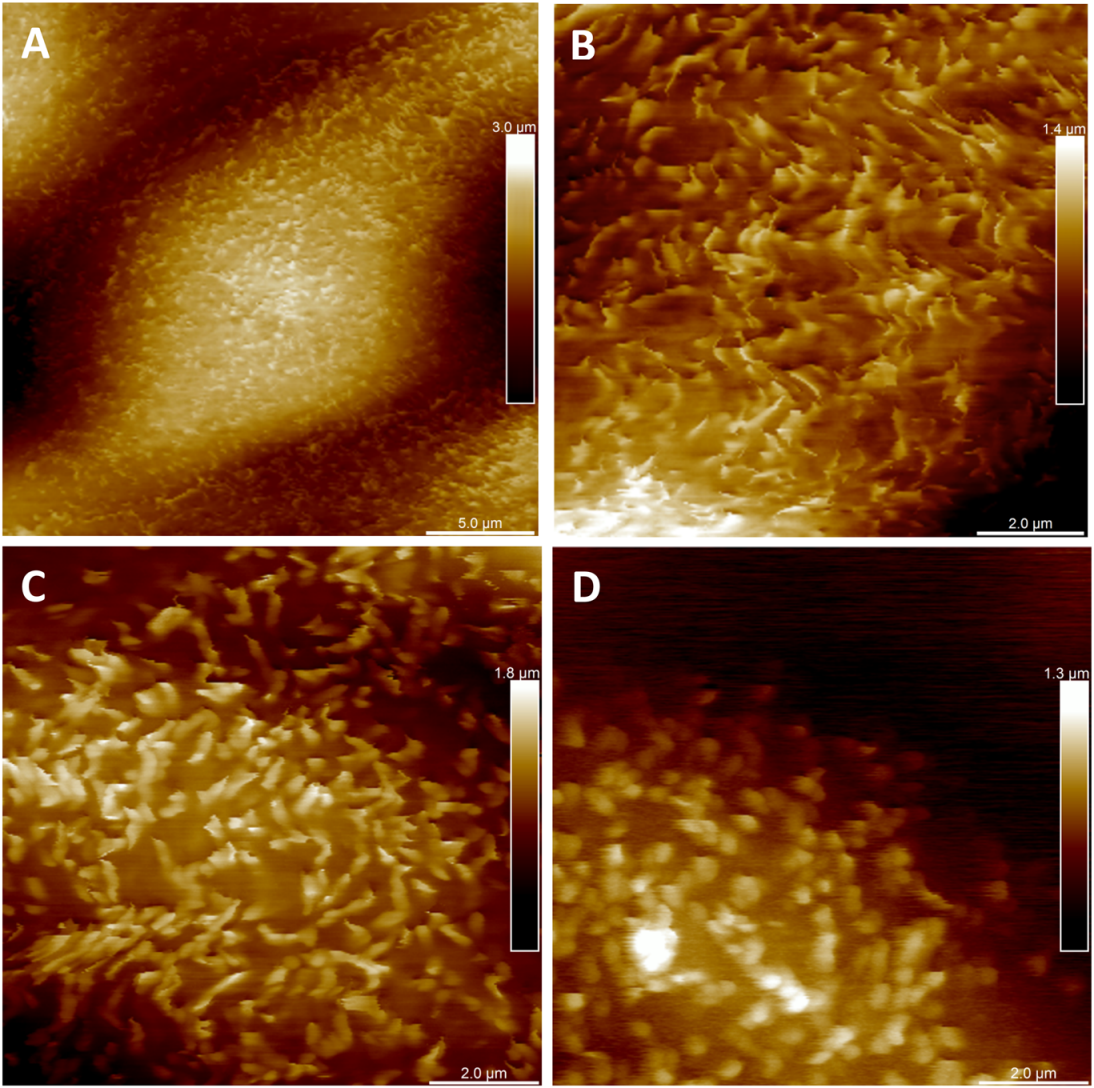
PeakForce Tapping image of a MDCK C11 cell, 25 x 25 µm (A) and 10 x 10 µm (B) scan with a vertical force of 150–250 pN. 10 x 10 µm scan of the same cell shown in B with a vertical force of 100–130 pN (C) (previously published as cover image in Microscopy and Analysis Jan/Feb 2015). Same area as shown in C scanned with a vertical force of 80–100 pN force (D).

Confluent monolayers of well‐differentiated MDCK C11 cells are not uniform in height and shape. Such height differences usually pose a problem for AFM scanning because most AFM probes have tip lengths below 5 µm. This causes shadowing and blind spots in the images where the tip was not able to reach the surface and the cantilever comes into contact with the cell body. The PFQNM‐LC probe, specifically designed for live‐cell PeakForce Tapping mode operation, has a 45‐µm‐long cantilever with a 17 µm long tip. The end of the tip has a length of 0.8–1 µm, with a controlled radius of 65 nm and an opening angle of 15° (Figure 1). This tip geometry avoids shadowing and significantly reduces the squeeze layer effect, thereby enabling imaging of cell surfaces even with large height differences. In a first experiment, MDCK C11 cells were fixed with 0.5% glutaraldehyde and imaged in PeakForce Tapping mode (Figure 2). Cells appear with typical dimensions, with both cell bodies of individual cells and cell–cell contacts clearly visible (Figure 2A). Scanning an 8 × 8 µm area indicated in Figure 2A revealed microvilli with lengths of 0.8–1 µm and diameter of 80–100 nm laying across the cell surface (Figure 2B). Even though the appearance of microvilli on life cells is not clear, an upright, brush‐like spatial configuration is likely, and fixing the cells destroys this native structure (Rother *et al*., 2015). Live MDCK C11 cells are shown in Figure 3 in which a 100 × 100 µm area of the monolayer was imaged with a peak force of 200 pN showing no disturbances by cantilever–cell contacts even though height differences of more than 10 µm are present (Figure 3A). Cell–cell contacts are clearly visible in the height and error image of these living cells (Figure 3B). The scan resolution (pixel size of 260 nm) was not sufficient to identify microvilli. At higher resolution as shown in Figure 4A (pixel size of 65 nm, scan speed 0.3 Hz (tip velocity 16.69 µm/s)), individual microvilli were resolved on the cell surface. Microvilli appeared as filaments pressed down to the cell surface because of the peak force of 150–250 pN. To improve the image resolution, the scan area was focused to a 10 × 10‐µm region on the center of the cell surface (pixel size of 26 nm), and data were obtained at a scan speed of 0.3 Hz (tip velocity 6.73 µm/s). The resulting image (Figure 4B) still did not show the cylindrical shape of microvilli but exhibited structures comparable to a waving grain field. As such, we believe that the applied forces were still too high and microvilli were still being displaced by the probe. A reduction of the peak force down to 100–130 pN at 0.2 Hz (tip velocity 4.41 µm/s) revealed individual microvilli with an almost cylindrical shape with roughly a length of 1 µm and diameter of 200 nm (Figure 4C). Decreasing the probe–sample interaction time by increasing the scan speed to 0.45 Hz (tip velocity 9.96 µm/s) reduced the totally applied vertical force to 80–100 pN to the tips of the microvilli. Under these imaging conditions (Figure 4D, showing the same region as 4B and 4C), only the upper part of the microvilli appear, exhibiting a mean height above the background of 332 ± 85.6 nm and a diameter of 341 ± 66.3 nm (mean ± standard deviation, *n* = 37).

## Discussion

**Figure 5:**
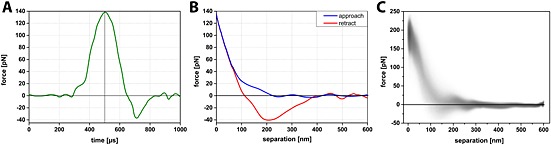
PeakForce Tapping data of a single pixel extracted from Figure 4B after background subtraction and baseline fitting displayed as force versus time (A) and force versus separation curve (B). Density plot (C) displaying in a gray scale the congruence of all force curves of Figure 4B (65 536 force curves).

**Scheme 1:**
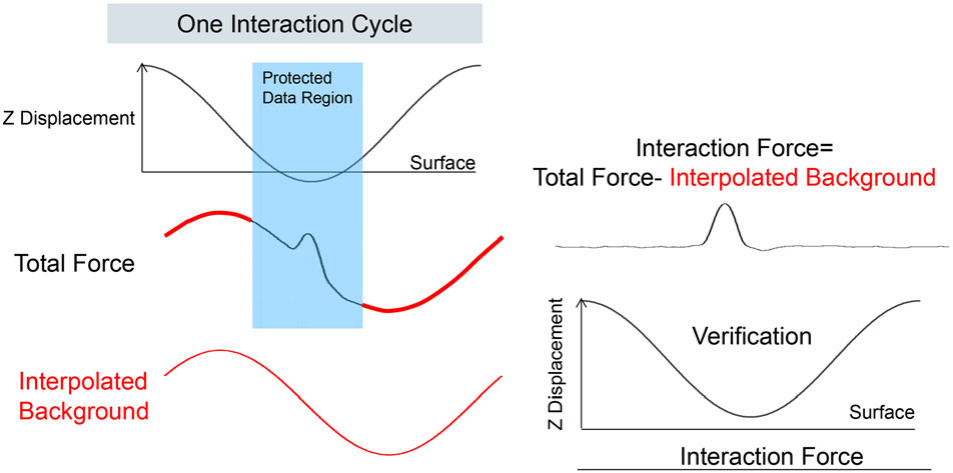
Scheme of the background subtraction algorithm. The sinusoidal Z modulation creates sinusoidal background. The algorism covers the region that contains the interaction force as protected data region. Subtracting the interpolated background from the total force recovers the interaction force.

Resolving high‐resolution structures on live cells by AFM requires control of the tip–sample interaction at low force in order to avoid cell damage and topographical alterations that may be caused by probe‐mediated deformation. The contact between the AFM probe and the cell surface causes a repulsive force, which deforms the membrane and bends the cantilever and provides the feedback signal to adjust the tip–sample distance by a piezo actuator. In contact mode AFM, an electronic feedback loop keeps the distance and therefore the force constant (Hansma *et al*., 1988; Hansma and Pietrasanta, 1998; Sokolov, 2013). Imaging soft and flexible structures like microvilli and cilia in contact mode is especially challenging because they are very soft and easily displaced in *x*, *y*, and *z* directions because of the high vertical and lateral forces applied by the probe resulting in poorly resolved structures in the AFM image. A way to reduce lateral forces is to oscillate the probe vertically at high frequencies during scanning, a technique known as TappingMode AFM. In this intermittent contact imaging mode, the probe–sample contact is limited laterally and temporally because of the vertically oscillating probe approach, which reduces friction and therefore minimizes potential dragging and pushing of flexible cell structures. In conventional TappingMode, the probe is vertically oscillated near the resonance frequency of the cantilever (tens to hundreds of kilohertz). Changes in the oscillation amplitude of the cantilever are used as the feedback signal for the *z*‐piezo feedback loop (Putman *et al*., 1994). In this mode, the probe–surface interaction force is controlled indirectly by averaging a large number of probe–sample interactions due to the high oscillation frequency. The tapping feedback signal (i.e., amplitude) reflects an averaged measurement of interaction forces across the entire range of vertical motion during the oscillation cycle, that is, not just the interaction at the bottom turning point. Force control is much more difficult than in contact mode because of the inherent instability of the feedback situation. The time constant of the cantilever resonance imposes limits on the feedback loop and can lead to large transient forces with possible tip or sample damage on rough surfaces. Additionally, the resonance behavior of the cantilever depends strongly on the sample properties such that images acquired on a mechanically heterogeneous sample using a constant amplitude set point at fixed frequency do not, in fact, reflect a constant interaction force. As such, while reducing the potentially damaging lateral force associated with contact mode, TappingMode AFM is unable to assure a constant imaging force – which is extremely important to imaging soft, delicate surface structures. PeakForce Tapping is an AFM mode in which the *z*‐position is modulated by a sine wave and the cantilever oscillates far below its resonance frequency. This reduces unwanted effects caused by dynamics of a resonating system, and even more importantly, for each probe–sample contact, a force–distance curve is used to control the probe–sample interaction force (Figure 5). The maximum loading force (peak force) of individual force–distance curves is used to adjust the *z*‐piezo position and thus keep the probe–sample interaction force constant. Essentially, the PeakForce Tapping mode is performing very fast force curves. Instead of the typical triangular Z waveform in the force curve, PeakForce Tapping uses the sinusoidal Z waveform. PeakForce Tapping uses the instantaneous direct peak interaction force as feedback. However, when PeakForce Tapping mode is used in liquid, there are large hydrodynamic forces due to the cantilever movement. As a fairly large Z modulation (larger than 600 nm peak to peak) is needed to pull the probe from contact with the very soft and sticky live cell, the hydrodynamic forces can be as high as 10–20 nN. Live cells can also have large topographical variations of up to a few microns in height. This means that not only will the cantilever distance to the sample vary as the AFM probes scan over the surface of the cell, but as a result of a squeeze film effect, the hydrodynamic forces acting on the probe will also change during scanning. Several technical approaches have been implemented to resolve this hydrodynamic force problem. First, the new PFQNM‐LC probe with a cantilever and tip geometry (shown in Figure 1) is designed to reduce the hydrodynamic force to around 2 nN. Second, a 17‐µm‐long tip moves the cantilever far from the sample surface and thus reduces the effect of the hydrodynamic force variation. Third, because of the dynamic nature and low stiffness of the cells, this often results in unsubtracted, residual background remaining in the resulting force curves. The new live‐cell background subtraction algorithm, which we applied to the captured force curves, removes this residual background and reveals the real applied vertical force. As shown in Scheme 1, in PeakForce mode, the Z modulation is sinusoidal. This creates a sinusoidal background to the cantilever deflection and the measured interaction force. The algorithm will treat the region that contains the interaction force as protected data region. This area of the curve will remain unaltered, while the algorithm fits the measured force outside the protected data region with a sinusoidal signal to determine the background signal. The algorithm then subtracts this background from the measured force to restore the true interaction force. By removing this background, a dramatic increase in signal to noise (background) is gained, which increases the sensitivity at which the peak force event is recognized even when caused by deviations from attractive van der Waals interaction below the baseline. This allows imaging at very low forces, which in turn is crucial for obtaining high‐resolution data on soft samples. In addition to dealing with the hydrodynamic forces, PeakForce Tapping mode also has an automatic gain control algorithm that controls and adjusts the imaging gain value during scanning by measuring the high‐frequency oscillation caused by the feedback loop (ScanAsyst‐Cell). As live cells are much softer than the stiffer underlying substrate (polystyrene, glass, etc.) on which they are imaged, they can sustain imaging gain values up to 10× higher than the substrate. The regular ScanAsyst auto gain control used in PeakForce Tapping mode is too slow to increase the gain on live cell and decrease the gain on the substrate at rates that allow accurate tracking of the AFM probe along both the cell and substrate surfaces. However, the ScanAsyst‐Cell auto gain increases the feedback speed for adjusting the gain value by 2× and enables the use of high gain values when imaging the surface of a cell and low gain values as the tip moves from the cell and onto the substrate. Together with the PFQNM‐LC probe, we can now reliably conduct PeakForce Tapping at modulation rates of up to 1 kHz on living cells. The total feedback bandwidth is now increased 16× because of the new ScanAsyst‐Cell auto gain and the increase of drive frequency. Together, this allows imaging of live cells at scan sizes of 100 × 100 µm at reasonable scan rates with high resolution. High‐resolution imaging at very low forces is shown in Figure 4. Although lateral forces are minimized, the vertical force of about 200 pN is sufficient to displace microvilli, leaving the impression of a waving grain field in the resulting image (Figure 4B). Individual microvilli appeared when the applied vertical forces are around 100 pN. This peak force is small enough to avoid displacement of microvilli as shown in Figure 4B. The level of distortion by the probe–sample interaction is clearly reduced, allowing accurate determination of the length and diameter of the microvilli at 1 µm and 200 nm, respectively, as well as their surface density (Figure 4C). However, even at this low vertical imaging force, there is still deformation of the microvilli as they did not appear as brush‐like upright cylindrical structures. This 10 × 10‐µm image was scanned at a scan speed of 0.2 Hz (tip velocity 4.41 µm/s). Increasing the scan speed to 0.45 Hz (tip velocity 9.96 µm/s), without changing the set point, lets microvilli appear as upright cylindrical structures (Figure 4D). Because PeakForce Tapping records force–distance curves for each image pixel, it was possible to determine the effectively applied force for each setting. Although the peak force set point was not changed, we determined that Figure 4C has a real vertical force of ~100–130 pN and Figure 4D has a real vertical force of ~80–100 pN. The real applied force depends on probe sample interaction and is more pronounced at forces close to the cantilevers force resolution (~15 pN for the used cantilever according to the equipartition theorem). The force range presented here was determined using density plots of an overlay of all curves in an image. Figure 5A and 5B shows PeakForce Tapping data of a single pixel extracted from Figure 4B after background subtraction and baseline fitting. It displays a force versus time (A) and a force versus separation curve (B). The density plot (C) displays the congruence of all force curves of Figure 4B (65 536 force curves) in a gray scale. This density plot reveals that the vast majority of the force curves show that the real applied vertical force is in the range of 150–220 pN. Obviously, increasing the tip velocity decreased the applied vertical force slightly, and we assume that this reduction of force below 100 pN (approximately 25% less force than applied in Figure 4C) enabled us to image microvilli as upright cylindrical structures. Interestingly, the cell surface itself was not resolved in these images, and the size of microvilli (length 332 nm and diameter of 341 nm) was below the values determined in Figure 4C. It is conceivable that due to very low forces, the probe cannot overcome the squeeze liquid layer and reaches the peak force too early. It is likely that the shape of the probe caused this effect as the end of the PFQNM‐LC probe has a length of 0.8–1 µm, and the transition from this end of the tip to the larger part of the probe entails broadening of the probe curvature. The microvilli layer is penetrated by the small part of the probe, and the approach causes squeezed layer damping between microvilli tips and the broad curvature of the probe. Furthermore, the feedback loop is also affected by the interaction between the cantilever and the cell surface. Given the abrupt change in topography between the top of the cell (lower left part image of Figure 5C) and the lower part of the cell (upper right part image of Figure 4C), it is very likely for the feedback loop to be challenged. As a result, these areas appear as unstructured and almost uniform pseudo‐surface between the microvilli.

## Conclusion

Atomic force microscopy probe–sample interactions can affect and modify soft and flexible samples by displacement and deformation. Imaging soft structures such as microvilli on living cells requires precise vertical force control down to the low pN range without attendant lateral dragging. Using an improved PeakForce Tapping background subtraction algorithm, in combination with the new PFQNM‐LC probe, has enabled the visualization of brush‐like microvilli in the native upright position by sensing the local peak interaction at the tip directly, at imaging forces approaching the cantilever's thermal noise.

## Conflict of interest

Izhar Medalzy, Shuiqing Hu, Andrea Slade, and James Shaw are employees of Bruker Nano Surfaces, Santa Barbara, CA, USA. Bruker Nano Surfaces Inc. is a manufacturer of scanning probe microscopes.
